# Perception of the difference between past and present stimulus: A rare orientation illusion may indicate incidental access to prediction error-like signals

**DOI:** 10.1371/journal.pone.0232349

**Published:** 2020-05-04

**Authors:** Robert Staadt, Sebastian T. Philipp, Joschka L. Cremers, Jürgen Kornmeier, Dirk Jancke

**Affiliations:** 1 Optical Imaging Group, Institut für Neuroinformatik, Ruhr University Bochum, Bochum, Germany; 2 Institute for Frontier Areas of Psychology and Mental Health, Freiburg, Germany; 3 Department of Psychiatry and Psychotherapy, Medical Center, University of Freiburg, Freiburg, Germany; 4 Faculty of Medicine, University of Freiburg, Freiburg, Germany; Psychologische Hochschule Berlin, GERMANY

## Abstract

A popular model for sensory processing, known as predictive coding, proposes that incoming signals are iteratively compared with top-down predictions along a hierarchical processing scheme. At each step, error signals arising from differences between actual input and prediction are forwarded and recurrently minimized by updating internal models to finally be “explained away”. However, the neuronal mechanisms underlying such computations and their limitations in processing speed are largely unknown. Further, it remains unclear at which step of cortical processing prediction errors are explained away, if at all. In the present study, human subjects briefly viewed the superposition of two orthogonally oriented gratings followed by abrupt removal of one orientation after either 33 or 200 milliseconds. Instead of strictly seeing the remaining orientation, observers report rarely but highly significantly an illusory percept of the arithmetic difference between previous and actual orientations. Previous findings in cats using the identical paradigm suggest that such difference signals are inherited from first steps of visual cortical processing. In light of early modeling accounts of predictive coding, in which visual neurons were interpreted as residual error detectors signaling the difference between actual input and its temporal prediction based on past input, our data may indicate continued access to residual errors. Such strategy permits time-critical perceptual decision making across a spectrum of competing internal signals up to the highest levels of processing. Thus, the occasional appearance of a prediction error-like illusory percept may uncover maintained flexibility at perceptual decision stages when subjects cope with highly dynamic and ambiguous visual stimuli.

## Introduction

Visual input is permanently changing and ambiguous. This puts demands on perceptual decisions to be fast and coherent, performing a tightrope act between stability and flexibility. However, continued visual stimulation and detected regularities therein allow predictions, and consequently, promote transmission of attenuated error signals given that top-down predictions are iteratively reconciled with bottom-up input in a hierarchical recurrent network [1–4]. Hence, the observation that repeated visual stimuli lead to reduced cortical activation during context-dependent cognitive tasks involving expectations and attention (for review see [5]) has been interpreted as an outcome of "predictive coding" at various stages of processing [4,6–13]. While it remains an open question whether reduction of visual responses to repeated or prolonged input may strictly emerge from computation of repetition probabilities [7] rather than arising from specific local adaptation dynamics [14–16], it has frequently been shown that abrupt alterations in temporal sequences of sensory input produce change-related signals that are generated “automatically” at early steps of cortical processing and that are typically labeled as prediction errors (for review see [17]). Basic forms of predictive coding have been proposed to exist already at earliest steps of visual processing in the retina [18] and LGN [19,20] where, in the framework of information theory [21], center-surround antagonism within receptive fields allow reduction in redundancies by removing predictable components of visual input. Mechanistically, exploiting the spatial (or temporal) correlations within natural images, the surround generates a statistical prediction of the signal at the center that is subtracted from the actual center signal to protect against intrinsic noise [18]. This type of predictive coding was refined for visual cortical neurons using a hierarchical model in which residual errors (signaling the difference between an input signal and its spatiotemporal prediction) propagate via feedforward connections and are compared at each level to correct its current estimate and then update feedback predictions, thereby iteratively decorrelating (i.e., whitening) actual input based on statistical regularities of natural scenes [1]. However, apart from detailed modeling mechanisms, the temporal constrains of predictive coding and to which extent residual error signals are passed through the visual hierarchy are less understood. Here we used temporal sequences of two stimuli comprising gratings with different orientations. Followed in rapid succession these stimuli have been shown to generate robust “prediction error-like” signals in primary visual cortex of anesthetized cats [15], thus, producing responses without influence of higher level expectation [22]. In the present study we investigated, whether we can find indications of residual prediction errors at such fast time scales in human perception.

## Materials and methods

### Participants

In total, 15 volunteers (age range: 18–26 years) participated in this study. Participants reported normal or corrected to normal vision (as further verified by the Freiburg visual acuity test (FrACT, [23]). They were naïve to the hypotheses and goals of the experiment (instructions and the task were introduced in a written form) and paid for their participation. All participants provided written informed consent prior to the start of the study. The study was approved by the local Ethical Committees of the University of Freiburg and carried out in accordance with the Declaration of Helsinki.

### Visual stimuli

Experiments were set up and run using custom-written Python scripts. Stimuli were displayed on a TFT screen (NEC, Model PA241W-BK) and viewed at 60 cm distance. Stimuli were circular windowed sine wave gratings (size = 4.8°, spatial frequency = 2.34 cycles/°) presented on dark background (0.4 cd/m^2^) with peak luminance of 376 cd/m^2^. The test stimulus was a windowed sine wave grating (size = 4.8°) presented in pseudo-randomized order at various orientations (-90° to 90° in steps of 15°) with spatial frequency of 1.28 cycles/° and peak luminance of 0.8 cd/m^2^.

### Procedure

**Fig 1:**
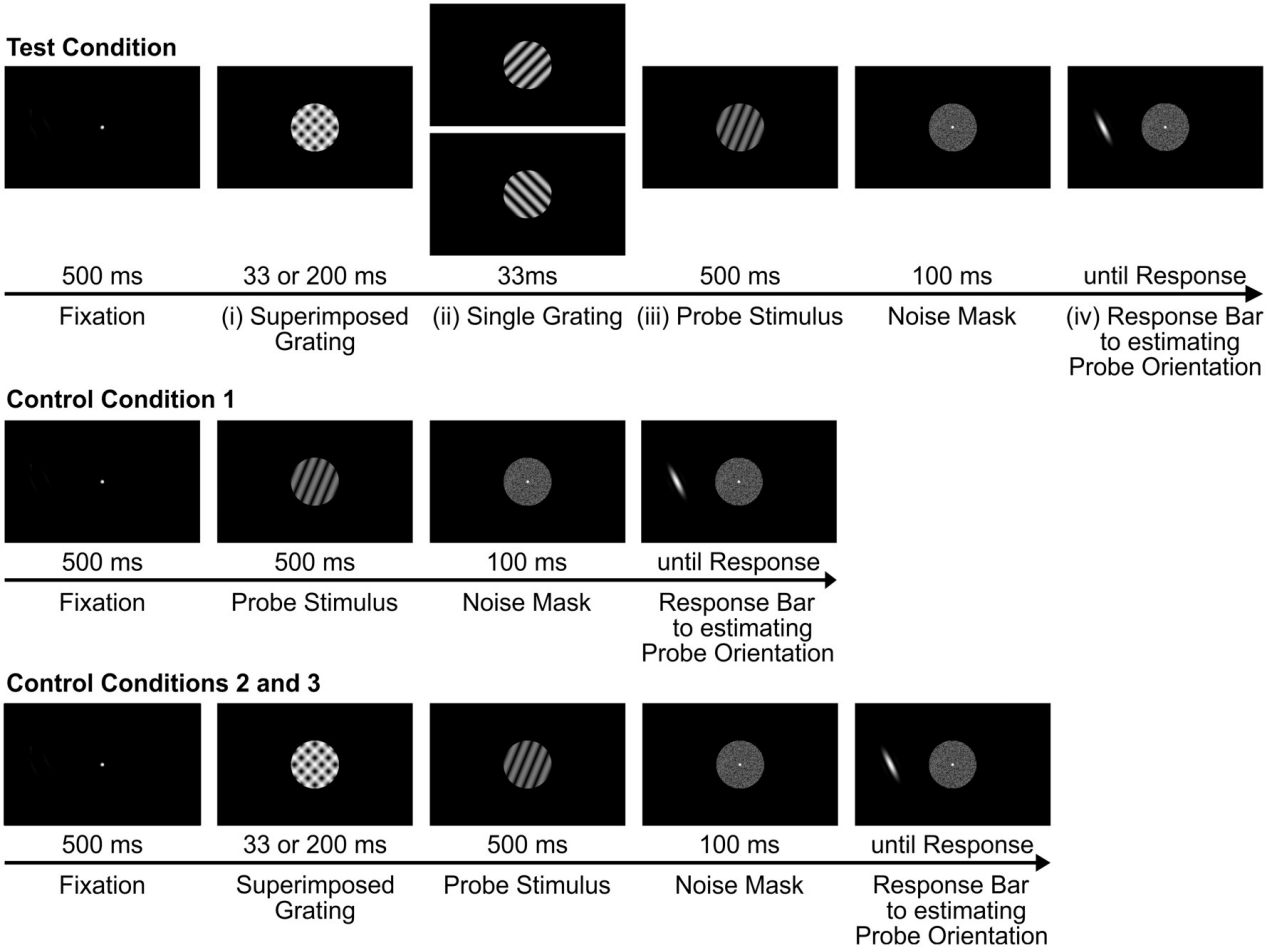
Schematic presentation of the paradigm used in this study. Test condition: After fixation, (i) superimposed gratings were shown for 33 or 200 ms followed by (ii) a single grating of either -45° or +45° orientation (i.e., matching to one of the orientations shown in the superposition before, size of stimuli = 4.8°). (iii) Thereafter, a probe stimulus was presented for 500 ms (iv.) Participants were asked to rotate a response bar until it matched the perceived orientation of the previous probe stimulus. Control conditions: In “Control Condition 1” only the probe stimulus (iii) was presented after fixation. In two further controls the superposition was presented at both durations, while omitting subsequent presentation of the single grating stimuli (ii).

The experiment consisted of 3 control conditions and 2 test conditions. After a brief acoustic signal, a fixation dot (Gaussian, FWHM: 0.17°) appeared in the center of the screen for 500 milliseconds. Control conditions: In one (“pure”) control condition, to measure baseline performance, a probe stimulus (Fig 1iii) was presented directly after the fixation dot for 500 milliseconds. In two further control conditions, gratings of oblique orientations were superimposed and presented with different durations, for either 33 ms or 200 milliseconds (Fig 1i). Test conditions: Superimposed gratings (with the same above 2 durations, presented in separate blocks) were followed by a single grating (Fig 1ii) comprising one of the orientations present in the preceding superposition (+45° or -45°, in randomized order). Just as in the pure control condition, in all conditions a probe stimulus appeared (Fig 1iii) followed by a mask (windowed white noise). After 100 ms a response bar was shown (Fig 1iv, single cosine wave, spatial frequency 1.07 cycles/°, in a Gaussian envelope, FWHM 2.07°) at one of 8 random locations next to the mask (distance to fixation point = 6.2°). Participants were requested to rotate the bar using a dial until the bar matched the perceived orientation of the test stimulus. After the response the next trial started.

In each experimental condition 13 different probe orientations were tested. Each such trial was repeated 8 times (i.e., subjects performed 104 perceptual decisions per condition). Participants were instructed to respond as fast and accurate as possible, while keeping fixation of the dot for the duration of each trial.

## Results

After fixation (Fig 1, top), human participants (n = 15) viewed a superposition of two orthogonal gratings (Fig 1i) for either 33 ms or 200 ms presentation time. The superposition grating was then followed by a single grating comprising one of the previously superimposed orientations (Fig 1ii, -45° or +45°). Thus, participants viewed a simple switch from a superposition grating to a single grating with two different time intervals. Thereafter, a probe stimulus, containing a low-contrast grating with a randomly chosen orientation was presented (Fig 1iii) succeeded by a noise patch. The participants’ task was to adjust a response bar (Fig 1iv), such that it matched the orientation of the previously perceived probe stimulus. We focused on whether and how the preceding superposition grating and/or the two orthogonal gratings influenced the percept of the probe.

**Fig 2:**
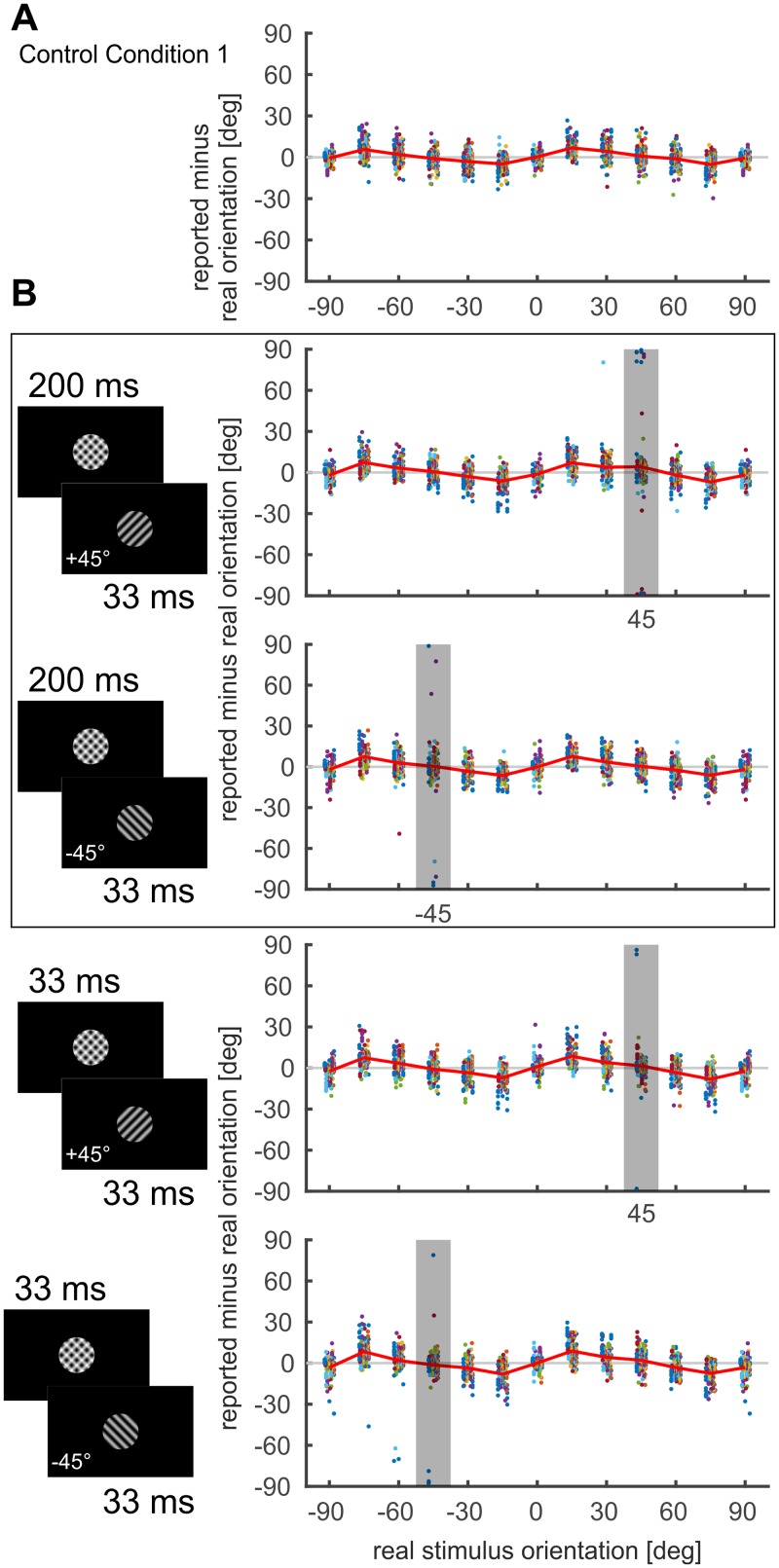
Incidental illusory percepts represent the difference between past and present stimulus. (A) Control condition 1, in which only the probe stimulus was shown. (B) Test condition (superimposed gratings followed by gratings with a single orientation). Outlined panels (black box) depict data for conditions in which superimposed gratings were presented for 200 ms, the lower two panels show data for shorter (33 ms) presentation time of the superposition. Gray bars mark orientations where the probe stimulus was identical to the single grating orientation (see icons at left of each panel). In each plot red lines depict mean, colored dots represent a single participant’s judgments for a given orientation of the probe stimulus for each of 8 trials. Probe orientations oblique to the single gratings were measured twice and are separately depicted as +/-90°. Note that multiple individual adjustment responses were orthogonal to the probe stimulus orientations (= illusory percepts; see particularly the gray rectangles in runs with 200 ms of the superimposed gratings).

First we describe baseline performance for the probe stimulus alone, i.e., without preceding gratings (Fig 1, “Control Condition 1”). Overall, perceived orientations matched the presented orientations with small biases away from cardinal axes (maximum mean bias value 6.8° ± 6.2°*SD* over all participants, Fig 2A). Such biases [24] were reported to be particularly strong at intermediate stimulus contrasts [25] and were here consistently found across all conditions (Fig 2A/2B), including additional control conditions, where solely the superimposed gratings were shown with different timing (S1 File of S1 Fig).

**Fig 3:**
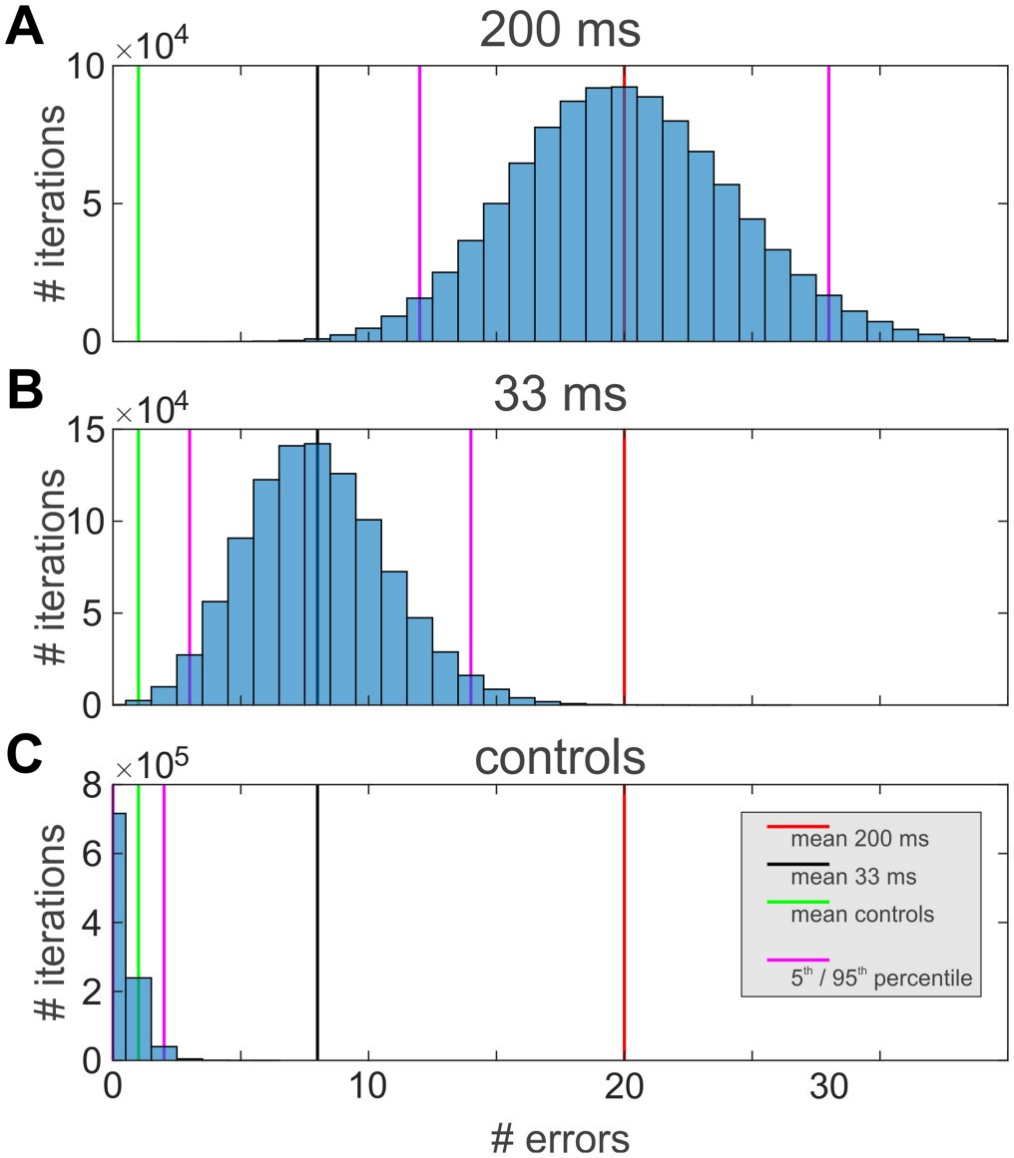
Incidence of illusory percepts was significantly different across all tested conditions. Illusory percepts were treated as errors, defined as judgments that deviated in orientation angle more than 4*SD* from the mean of the density distribution obtained for control conditions. For each condition, data for the +45° and -45° probe orientations were randomly resampled (10^6^ iterations). (A) Error count distribution for the condition with superimposed gratings presented for 200 ms followed by grating with single orientation. Mean error count = 20 (red line). Probability to obtain 8 error counts (black line), p = 0.0015. (B) Same as (A) for the test condition where the switch from superposition to single orientations occurred after 33 milliseconds. Mean error count = 8 (black line). Probability to obtain 20 error counts (red line) or more, p = 0.00022. Probability to obtain 1 error count (green line) or less, p = 0.00026. (C) Across all participants, all trials, and all three control conditions (15 subjects, 8 trials, 3 conditions, 2 orientations of the probe stimulus (cf. gray areas in Fig 2), in total 720 cases) one error as defined above occurred (green line). Consequently, resampling of control conditions shows that the chance to obtain error counts in the range as found for the switch from superimposed to single gratings (see A and B) was zero. In all plots purple lines indicate 5^th^ and 95^th^ percentile.

**Fig 4:**
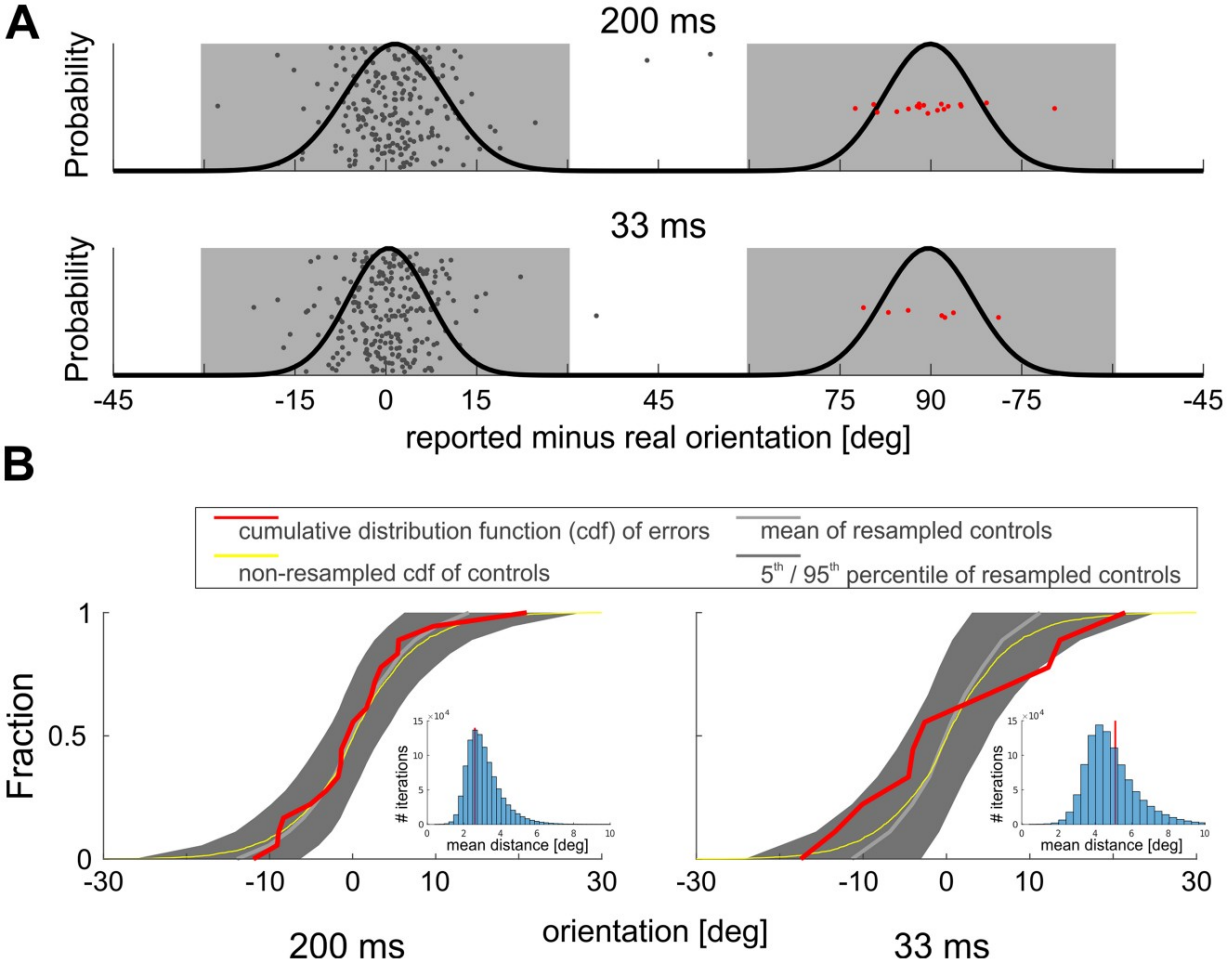
Illusory orientation percepts have the same distribution as percepts of true orientations. (A) Illustration of judgments around true orientation (gray dots) and illusory percepts (i.e., errors, reported orientations orthogonal to the actually presented probe orientation, indicated with red dots) for both test orientations -45° and +45° (centered at 0°, n = 240 data points in each upper and lower panel) with fitted normal distributions (black curves). Vertical offsets of data points are to avoid overlap and are only for demonstration purposes. (B) Cumulative distribution functions (cdfs) of perceived errors (only cases within 4*SD* around 90° were used, see red dots in (A) within gray shaded areas; n = 18 for 200 ms, n = 7 for the 33 ms condition) and resamples of control conditions using the same number of cases per sample (18 and 7, respectively, see Methods). Red curves depict cdfs of errors. Gray line depicts mean and gray shading represents 5^th^ and 95^th^ percentile boundaries of resampled controls. All distributions were centered at 0°. Note that the cdf of all errors is within percentile boundaries of resampled controls except for one case in the 33 ms condition. Histogram insets show mean absolute distance of each resample from the non-resampled (“reference”) cdf of controls (yellow curve). Red line indicates the mean distance of errors from the reference cdf. Chance to obtain a resample with distance equal or greater than perception of errors: p = 0.668 for the 200 ms switch condition, p = 0.360 for the 33 ms switch condition. The graph shows that the error cases are not extreme values of a distribution of erroneous percepts (or percept variance) but represent a separate set of rare cases with illusory percepts orthogonal to the probe stimulus.

Surprisingly, in the test conditions, when the superimposed grating was followed by a single grating, a number of judgments clustered around orientations that were orthogonal to the to-be-matched probe orientation (Fig 2B, see “outliers” within gray bars and around nearby orientations). These “illusory percepts” of the difference between past and present orientations (which we refer to as perceptual errors) appeared in 7 of the 15 participants (dot colors in Fig 2 denote individual subjects) almost exclusively at probe orientations parallel to the single grating orientations present after the superposition grating (Fig 2, cf. gray bars; obtaining the illusory percept by chance in all 4 conditions: p<10^−6^, based on 10^6^ shuffles of data between different orientations). This indicates that the effect may involve specific adaptation to the orientation [26], that had remained present after the switch from superimposed gratings. Importantly, the effect was also critically dependent on switch timing: when the superimposed gratings were shown for 200 ms (Fig 2B, outlined panel), the probe stimulus at the adapted orientation was 20 times (8.3% of trials) perceived as being orthogonal (or close to orthogonal) to its real orientation, whereas this was only 8 times (3.3% of trials) the case for the faster switch after only 33 ms (Fig 2B, two bottom panels). This is consistent with recent cat studies which showed that the generation of error signals representing the difference between past and present orientations needs time to build up and seem to be reliably formed when switch times are larger than 33 ms [15,27]. In contrast, over all control conditions only two errors (defined as judgments 4*SD* outside the mean of the density distribution obtained for control conditions) occurred within a total of 4680 trials (S1 File of S1 Fig). The significance of these observations was evaluated by resampling trials of each condition at the respective +45 and -45-degree probe orientations (data points within gray bars in Fig 2) and counting the number of such errors (i.e., illusory percepts) across the different conditions (Fig 3). Further we found that the density distribution of illusory percepts was similar to the distribution of judgments around the true orientation (Fig 4, 33 ms p = 0.360, 200 ms p = 0.668, see S1 File of S1 Text), pointing to a systematic effect rather than to accidental occurrence of erroneous responses. Such a similar scatter of orientation judgments may therefore reflect shared processing resources of signals encoding the difference between past and present and signals encoding the real current input. The fact that both signals are generated in a stimulus regime of high temporal dynamics and low contrast without higher-order expectations favors primary visual areas as their common cortical origin [15].

## Discussion

We revealed cases of illusory visual percepts in which perceived orientations represented the arithmetic difference between orientations seen in the immediate past and actually present orientations. The illusory percept cannot be explained by known tilt-aftereffects of adaptation [28,29] and its rare appearance is incompatible with predictive coding schemes in which error (i.e., difference) signals are “explained away” [6] during recurrent processing steps. Instead, our observations suggest that error signals do not always diminish but sometimes remain accessible at late processing stages forming awareness [30]. The underlying neuronal difference signals may penetrate perception through post-perceptual decision, occasionally ignoring information about the actually present stimulus at higher hierarchy levels. Thus, our results may support the view that perception depends on late decision stages [31–34] at which multiple signals of recent stimulus history [31] are analyzed and integrated before reaching conscious awareness [35–38].

Interestingly, during free viewing of natural scenes intersaccadic durations in humans are on average 250 ms [39], thus, covering a similar time range as spanned by the superimposed gratings presented for 200 ms in our study. Note also that the illusory effect was less prevalent with shorter presentation time (33 ms) of the superposition. In fact, changes between low and high frequency stimulus regimes are proposed to allow for coarse-to-fine processing of visual information [40–48], providing an efficient strategy for the detection of salient structures in the environment [45]. Thereby, longer stimulus durations (on the time scale of a single fixation) facilitate identification of orthogonal orientations [49], while reducing redundancy near adapting orientations [50]. The rare appearance of the illusion in our study might reflect an occasional erratic overcalibration of such function.

**Fig 5:**
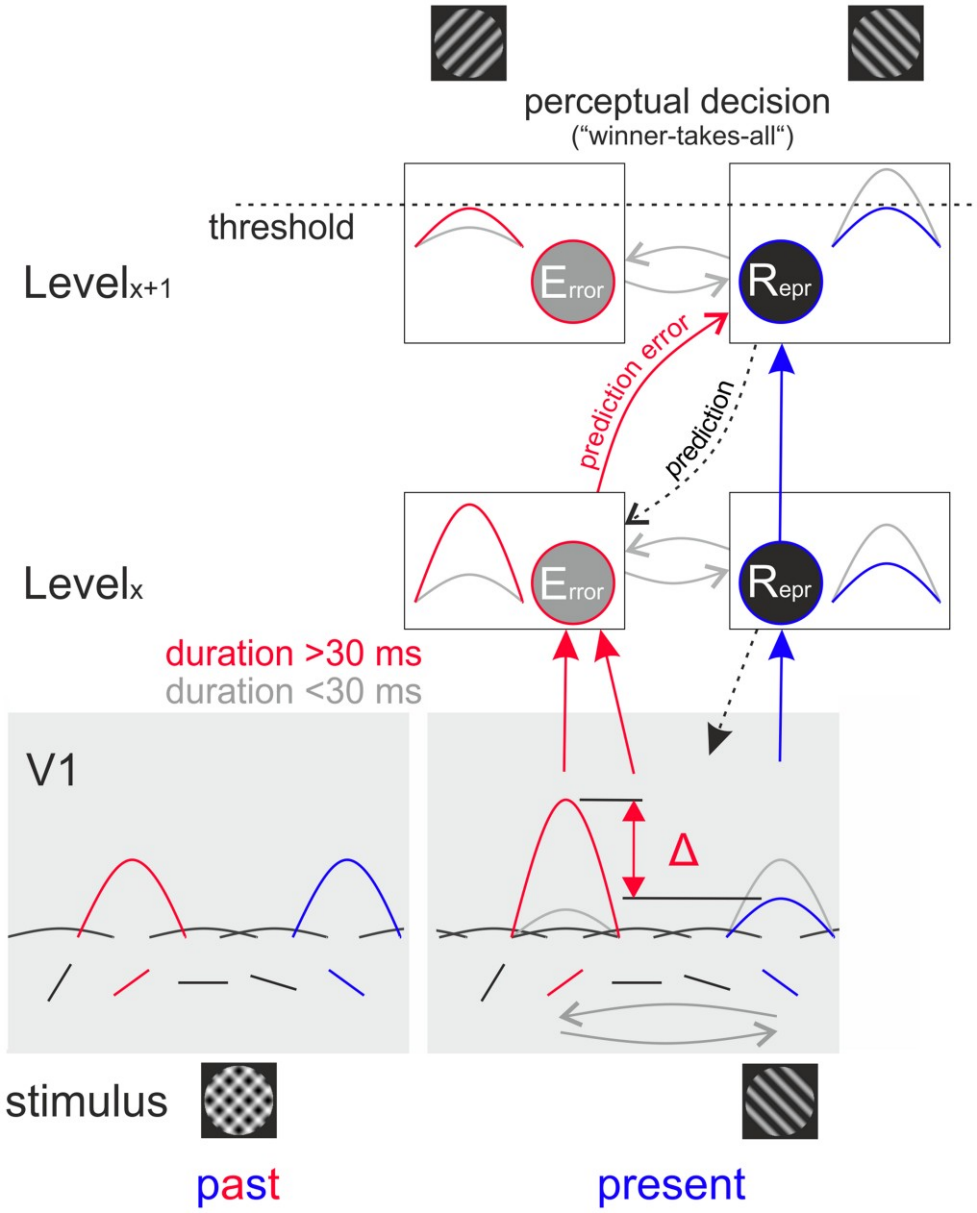
Illustrative cartoon: Generating illusory perception of the difference between past and present stimulus (“prediction error”) within a predictive coding framework. Presentation of the superposition of two gratings (stimulus past, shown bottom left) activated neurons in V1 that are tuned to the given orientations (red and blue peaks). If the superposition remains for more than ~30 ms, abrupt removal of one orientation facilitates a strong orientation-selective "off" response [15], while responses to the present (i.e., sustained) orientation undergo adaptive decrease (bottom right, cf. amplified red and reduced blue peak amplitudes, respectively; stippled black downward arrow indicates feedback). "Off" responses, or alternatively, the difference of the peaks’ amplitudes (Δ) are forwarded (straight red arrows) to error units ("Error", red framed circles, peaks at left represent their output) further downstream (Levelx). In parallel, representation units (“Repr”) carry adapted signals of the present orientation (blue peaks). Representation units at higher hierarchical levels (Levelx+1) receive bottom-up prediction errors (curved red arrow) and send predictions via top-down projections (dotted curved black arrow) that gradually downsize prediction errors (red peaks at left) in upstream and laterally connected (gray curved arrows) error units (for clarity only two hierarchical levels are depicted; see [1] for a detailed theoretical account and [17] for review). At a putative perceptual decision stage, signals from error units (top red peak) and representation units (top blue peak) compete with each other, potentially weighted and thresholded (stippled horizontal line), to finally form conscious perception. If signals conveyed by error units are of large amplitude (and not explained away, as in the case of left gray peaks), they might occasionally outplay signals from representation units (encoding the present stimulus). This gives rise to illusory perception of the difference between past and present stimulus (i.e., the removed orientation, top left). Note again that responses to the present orientation underlie V1 adaptation. In contrast to non-adapted responses (gray peaks at right), adaptively reduced amplitude in representation units (blue peaks at right) increases the chance of being outbalanced by error unit amplitudes (red peak at left).

Why is the illusion observed only occasionally? The exact causes of the observed rare cases of illusory perception of difference signals between past and present orientations remain open. Illusions are often revealed by artificial stimulus configurations that produce extremes of what the system has evolved to handle [51]. In contrast to studies that evaluate effect significance by comparing test and controls through comparison of the variance introduced by the treatment (a varied factor, assumed to affect the whole sample/population in a similar way) with the combined variances within the groups, we here derived multiple errors as "outliers" that, however, cannot be explained by chance. In particular, using permutation tests, we evaluated that the incidence of perceiving errors was significantly different across all tested conditions and that errors (i.e., illusory percepts) show the same distribution as judgments estimating true orientations (Figs 3 and 4, respectively), thus, ruling out simple outlier explanations. Moreover, the fact that across all control conditions illusory percepts were “practically non-existent” clearly suggests that the illusion depends on the introduced dynamics by the stimulus switches from superposition to single gratings, and hence, on the availability of the resulting difference signal at higher levels [52]. Our experiments in cats showed consistent generation of the difference signal in primary visual cortex with increasing switch time intervals [15]. This is in accordance with the present results and is most likely based on increasing impact of inhibitory suppressive mechanisms over time [15,16,53]. Thus, the fact that in the present human study the illusory percept occurred very rarely may straightforwardly depend on currently unknown impact of factors producing difference signals (such as stimulus durations, contrast, spatial frequencies) that vary in individual subjects or on psychometric factors (e.g., internal noise, attention) that influence individual perceptual decision processes during which signals of current stimuli compete with simultaneously present difference signals, Fig 5). A systematic exploration of factors enhancing the present illusion is therefore needed in future studies to identify the exact causes of its appearance in individual subjects.

### Potential relationships of our results to a predictive coding scheme

At a more abstract level, given the framework of predictive coding models [1–4,18,19], we propose that the illusory percept may originate from an internally generated prediction error that, in our case, becomes occasionally conscious without higher-order expectations [22,30].

Interestingly, also in human EEG recordings, changes in stimulus orientation evoked a deviant signal that was most strongly tuned to orthogonal orientations [12]. These signals were interpreted as correlates of a prediction error, coding the relative difference between predicted and observed orientations [12]. Note that in the current study we used switches from superposition to single gratings. This allows interpretation of the “deviant” error signal as a result of computing the arithmetic difference of subsequent stimuli (i.e., as the difference between past and present orientations) rather than resulting from a complete stimulus exchange. Our previous experiments in cat primary visual cortex [15,16] showed that after the switch from superimposed to single gratings, populations of neurons encode the present (sustained, and thus, adapted) orientation with suppressed amplitudes, whereas populations tuned to the removed orientation produce a strong and persistent [54,55] “off” response [15], as depicted in Fig 5 (cf. changes of peak activity at bottom [past vs. present], blue and red, respectively). Signals following stimulus removal are commonly referred to as visual off-responses, which tend to increase with stimulus duration [56]. Such off-responses are also found in the somatosensory cortex [57]. Consequently, within the same cortical region two functionally distinct populations co-exist, one suppressed and one containing enhanced iconic memory [58,59], capable of providing different information within predictive coding circuits [60]. “Off” responses may either directly signal “prediction errors” (i.e., reporting difference to past; Fig 5, left straight red arrow) or prediction errors may be read-out at subsequent stages (Fig 5, Level_x_) by computing the relative difference of amplitudes signaled by both populations [15] (Fig 5, right straight red arrows).

As suggested in classical models of predictive coding [1,4], prediction errors may propagate via feedforward connections (Fig 5, curved red arrow) and are compared at higher hierarchical levels (Fig 5, “Level_x+1_”, only one higher level is shown for clarity) that correct current estimates of the input signal and generate top-down prediction (Fig 5, black dotted curved arrow) to minimize residual errors (Fig 5, red and gray peaks at left sketch strong and weak residual errors, respectively). Finally, we hypothesize a perceptual decision process (Fig 5, top panel, horizontal stippled line illustrates perceptual threshold), which thresholds (or actively weights) input from residual error signals (peaks at left) and input from current stimulus signals (peaks at right). The occasional perceptual access to prediction errors may depend on internal noise of processes that produce perceptual decisions, possibly also involving attentional resources, or on computations responsible for generating (or sustaining) strong enough “off” responses (Fig 5, left red peaks). In subjects without illusory perception, the chosen stimulus contrast, the duration of the superposition stimulus, or other factors, may not be sufficient to produce the necessary strong “off” responses (left gray peaks) or fail to produce sufficient adaptation (right gray peaks). As seen in our data, short presentation times increase the tendency of encoding the current stimulus. Most likely because cortical "off" responses depend on suppressive processes [53] that need time to build up. Thus, in most cases signals encoding the current input (even though adapted) might be larger than signals carrying prediction errors (i.e., errors were sufficiently explained away, cf. left gray peaks).

Altogether, we present a most simple version of predictive coding, where persistent activity of previous input (i.e., prediction error-like signals) competes with adaptive response components of current input at fast time scales. The first coding step is based on our empirical results obtained in cat V1 [15], where we showed that removal of an orientation generates a tuned off response (Fig 5, red peak in bottom right pane), while responses to sustained orientations undergo adaptive decay (Fig 5, blue peak in bottom right panel). Accordingly, due to increased contribution of orientation-selective off-responses, the combination of adaptive- and off-response components results in a representation of the difference between the past and the present image, i.e., generating an error signal tuned to the removed orientation (Fig 5, cf. delta at bottom right). Error signals may then be reduced by feedback from higher processing levels (Fig 5, cf. peaks at left, Level_x_ and Levels_x+1_), as suggested by predictive coding models of visual processing streams [1]. Adapted signals tuned to the present orientation propagate in parallel up the hierarchy along representation units (Fig 5, cf. peaks at right). Finally, a perceptual decision is made by thresholding peak activity from error and representation units, resulting in a discrete decoding of either the present (Fig 5, top right) or the removed orientation (Fig 5, top left), where the latter signifies a difference (error) signal.

## Conclusions

We present a visual illusion in which observers perceive the difference between previously seen and actual orientations rather than the actual orientation itself. The illusion was produced by presenting the superposition of two orthogonally oriented gratings followed by abrupt removal of one orientation after either 33 or 200 milliseconds. However, the illusory percept occurred only rarely. Thus, apart from a clear dependence of the effect on the stimulus time interval, other factors driving this illusion remain to be found.

As the illusion reflects the arithmetic difference between a previously seen and a current stimulus we interpret our finding within the framework of predictive coding. Predictive coding provides decrease in redundancy [18] particularly suited to low noise situations [61], but bears the risk of snap judgments in case of uncertain input dynamics. From a general modeling point of view the low number of illusory percept cases found here might therefore reflect a signature of an in-build instability that prevents the system from running into potentially wrong attractors in ambiguous stimulus situations [62–66]. As demonstrated in recent simulations that coupled predictive coding schemes to intrinsic brain dynamics, such a computational strategy ensures that conditional expectation maintains uncertainty, which in turn allows for flexible representations of contingencies and ambiguities in the world [67]. As our experiments imposed neither specific demand on the task, nor provided additional contextual influence, further studies may unravel how the presently low frequency of the illusory percept varies with attention [11,12], with spontaneous activity [68], or with global changes in brain state [69]. We here propose a speculative scheme of predictive coding, where error signals, emerging most probably from first steps of cortical processing dynamics, are not explained away but maintained and pushed through the perceptual hierarchy to potentially benefit flexible context-dependent and time-critical post-perceptual decisions [31,70–75] at higher cortical entities.

## Supporting information

S1 File (PDF)

S1 Data (CSV)
