# Supplementary material for: Perception of the difference between past and present stimulus: A rare orientation illusion may indicate incidental access to prediction error-like signals
Source: PLoS One. 2020 May 4;15(5):e0232349. doi: 10.1371/journal.pone.0232349 (PMC7197803; doi:10.1371/journal.pone.0232349)
Supplement: S1 File — (PDF) [file pone.0232349.s001.pdf]

Supporting Information

1. Supplementary Figure

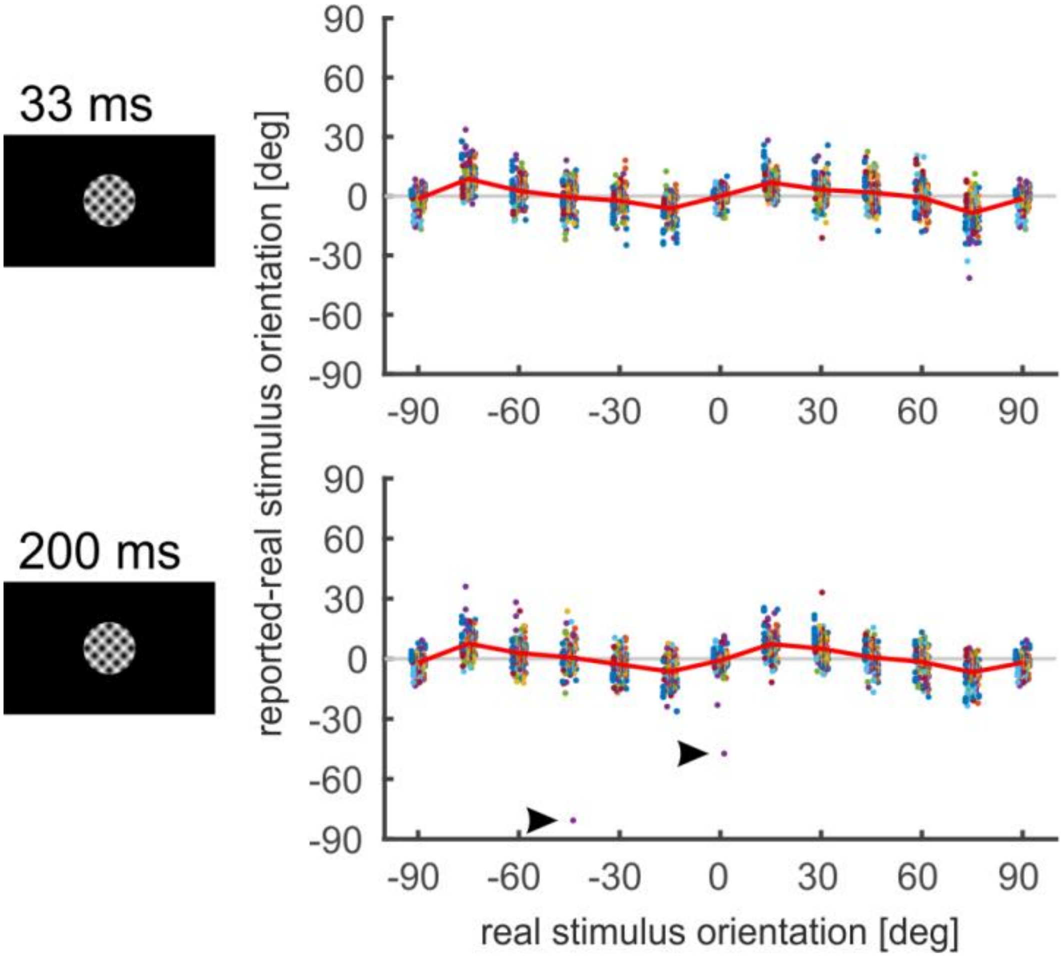

Supplementary Figure 1. Over all control conditions "prediction error" occurred only twice as illusory percept in 4860 judgments.

Control conditions in which superposition gratings were followed by the probe stimulus. In the upper panel data are shown for the condition where the superposition was presented for 33 ms, the lower panel shows data for 200 ms presentation time (see icons at left). In each plot red line depicts mean, dot colors represent a single participant's judgment for a given orientation of the test stimulus for each of 8 trials. Note that across all control conditions only two data points were 4*SD* outside the mean of judgments and only in one of these cases the test stimulus was perceived as being approximately orthogonal to its real orientation (lower panel, cf. purple dot at 0° and dot at -45°, respectively, marked with arrow heads).

2. Supplementary text

Distribution similarity test

To test the similarity of distributions of illusory percepts ("prediction errors") and non-errors, a resampling test was performed. First, a cumulative distribution of the errors (error cdf, Fig. 4B, red curves) was constructed. An error is defined as an orientation judgement >4*SD* from the mean found in each switch condition (Fig. 4A, red dots). Second, from all three control conditions a reference cumulative distribution function (reference cdf, Fig. 4B, yellow curves) was constructed. Third, 10^6 samples of n random cases were taken, with n equal to the number of errors tested (n = 7 for the 33 ms condition and n =18 for the 200 ms condition). For each random sample we calculated a cumulative distribution. Finally we calculated the mean and the variance across these 10^6 cumulative distributions to achieve a reference window (Fig. 4B, gray curves represents mean, shades represent 5th and 95th percentile) for comparison with the error cdf (Fig. 4B, red curves).

Next, the angle distance between each point of the error cdf (shifted by 90° to cluster it near 0° instead of +/-90°) and the corresponding point of the reference cdf was calculated (Fig. 4B, horizontal distance between red curve and yellow curve at each point on the red curve). To obtain a single value describing this deviation, the mean of all absolute distance values was calculated (Fig. 4B insets, vertical red lines). This procedure was repeated with the reference distribution and the individual distributions of the aforementioned 10^6 resamples of the control conditions (Fig. 4B individual resamples not shown but represented by their mean, 5th and 95th percentile, gray line and shades, respectively). Thus, a distribution of distances between resamples and the reference distribution (their own source distribution) was obtained (Fig. 4B, blue histograms in insets). If the illusory percept distance (Fig 4B, insets, red lines) is below the 95th percentile of this distribution, it or a more deviating distribution could have been obtained from the baseline distribution with a 5% or greater chance. Therefore, it would not be significantly different from the baseline distribution at the $p<0.05$ level.
